# Growth Performance, Blood Metabolites and Carcass Characteristics of Black Goat Kids Fed Diets Containing Olive Cake

**DOI:** 10.3390/ani14020272

**Published:** 2024-01-15

**Authors:** Belal S. Obeidat, Milton G. Thomas

**Affiliations:** 1 Department of Animal Production, Faculty of Agriculture, Jordan University of Science and Technology, P.O. Box 3030, Irbid 22110, Jordan; 2 Department of Animal Science, Texas A&M AgriLife Research, Beeville, TX 78102, USA; milton.thomas@ag.tamu.edu

**Keywords:** black goat kids, olive cake, carcass characteristics, meat quality, blood parameters

## Abstract

**Simple Summary:**

For many years, alternative feeds have been employed in place of traditional feeds to lower the expense of feeding animals and to lessen the environmental damage if the feed is not disposed of. Growing black kids were fed olive cake at 0, 75, and 150 g/kg. The diets’ chemical compositions were comparable. The cost of gain dropped when olive cake was used in the diets.

**Abstract:**

This study investigated the dietary effect of incorporating different levels of olive cake (OC) on the metabolic responses, growth performance, and carcass characteristics of black goat male kids. Thirty kids (body weight = 17.3 ± 0.40 kg) were randomly distributed into one of three equally sized dietary groups: a control diet (CON), OC at 75 g/kg (OC75), and OC at 150 g/kg (OC150) of dietary dry matter (DM). The results revealed that the intake of DM, crude protein (CP), neutral detergent fiber (NDF), and acid detergent fiber (ADF) was similar (*p* ≥ 0.11) among the three treatment groups. However, the OC150 group had the greatest (*p* < 0.0001) ether extract (EE) intake compared to the OC75 and CON groups. The growth measurements were similar (*p* ≥ 0.13) among the three groups. Feed efficiency was not affected by the inclusion of OC. In contrast to the CON diet, the cost of gain was, however, reduced (*p* = 0.004) in the OC diets. All three treatment groups’ digestibility of DM, CP, and ADF was similar. However, when compared to the OC75 and CON groups, the digestibility of NDF was better (*p* < 0.05) in the OC150 group. The N intake did not differ among the three experimental groups. Nitrogen retained as g/d was higher (*p* = 0.04) in the OC150 and OC75 groups compared to the CON group, while retention as a percentage (g/100 g) was similar among the three groups. Except for intermuscular fat, total fat, leg fat depth, and tissue depth, the inclusion of OC did not result in any discernible treatment effects on the carcass and meat quality parameters. Only alanine aminotransferase enzyme activity was lower (*p* < 0.0001) in OC-treated groups compared to the CON group. In summary, incorporating OC at 75 g/kg and 150 g/kg levels into the diets of black goat kids had positive comparable effects on some parameters related to growth performance, carcass attributes, and meat quality. Importantly, utilizing olive cake led to cost savings in production and may serve as a viable alternative feed source in goat nutrition.

## 1. Introduction

Goats have a pivotal role in Jordan’s agricultural sector, contributing significantly to the farmer’s food security and economic livelihood [1,2]. Goats are resilient and adaptable livestock that can thrive in harsh environmental conditions and diverse ecosystems. According to the most recent statistical summary in November 2022, the goat population was 803,940 head in Jordan [3], second only to sheep numbers, reflecting their importance.

Due to its arid and semi-arid regions, Jordan encounters various challenges related to its climatic conditions, water scarcity, and limited rangelands. The productivity of goats is hampered by many factors, including nutrition, health, and management practices, as well as the challenges posed by climate change, disease outbreaks, and limited natural resources [4,5]. However, feed shortage, high feed prices, and rangeland grazing limitations are among the most important problems limiting goat production [4].

The utilization of alternative feed resources in livestock nutrition has gained substantial attention due to their potential to enhance sustainability and reduce production costs. One such resource that has shown promise in recent years is olive cake (OC), a residue from the extraction of olive oil, which is a mixture of skins, pulp, stone wall, kernel, and oil residues [6] and accounts for one-third of the processed weight of olive fruit [7]. Awawdeh and Obeidat [8] reported that the utilization of OC at 100 g/kg in the diets of growing Awassi lambs had no impact on nutrient intake. Likewise, Abo Omar et al. [9] noted that incorporating 150 g/kg of OC had no detrimental effect on the performance of Awassi sheep. The intake of dry matter (DM) and additional nutrients in diets containing OC can vary, and it relies on the composition of the diets and the extent of the inclusion of OC. Obeidat [6] reported that adding 150 g/kg of OC to the diet of Awassi lambs could be a cost-effective dietary option compared to conventional feed sources that would not detrimentally impact animal performance.

The incorporation of alternative feed by-products into animal nutrition may be influenced by their source, quantity, and processing methods. We hypothesized that using a suitable level of OC in the diet of goat kids could enhance their growth performance, economic outcomes, and carcass qualities. This improvement is expected through increased feed intake, enhanced digestibility, better meat production and composition, more favorable blood metabolite levels, and a reduction in feeding costs, ultimately leading to increased profitability. Considering the details mentioned above, the objective herein was to evaluate how various levels of OC impact the performance, growth and health of black goat kids.

## 2. Materials and Methods

### 2.1. Study Design, Kids, and Diets

This study was conducted at the Agricultural Research and Training Unit of Jordan University of Science and Technology (JUST). The study area was characterized as semi-arid, located at 510 m above sea level and at a latitude of 32.30 degrees north. Thirty young male black goats, averaging 17.1 kg in weight and 90 days old, were randomly assigned to one of three different diets. There were three diets, as shown in Table 1: no OC (CON; n = 10), 75 g of OC per kilogram (OC75; n = 10), and 150 g of OC per kilogram (OC175; n = 10). Based on the NRC [10] guidelines, all diets were isonitrogenous, adjusted to provide 160 g/kg of crude protein of dietary DM, and tailored to meet the requirement of nutrients for growing male kids to gain 100 to 150 g/d. The trial spanned 73 days, with the initial 10 days designated for acclimating kids to pens and diets, followed by 63 days used for the collection of data. The kids were fed two times daily, receiving two equal meals at 9:00 and 15:00 h, and were housed in individual pens measuring 1.5 m × 0.75 m. No bedding material was provided in the pens throughout the study. Before diet formulation, OC was sourced from nearby olive oil mills, air-dried and dispersed, and subsequently ground to facilitate its thorough mixing with other dietary components and to prevent sorting in a total mixed ration.

The chemical composition of the OC was 76.5, 578, 354, and 101.3 g/kg on a DM basis for CP, NDF, ADF and EE, respectively. To ensure consistency in chemical makeup, the diets were thoroughly mixed every two weeks throughout the trial, and the samples were promptly collected right after mixing (Table 1). Throughout the study’s duration, all the young goats were provided with a complete mixed ration diet and had unrestricted access to fresh water through the use of 10 L plastic containers. Before each day’s feeding, any leftover feed was collected, weighed, and recorded to determine daily DM intake and other nutritional intakes, and representative samples were collected for subsequent chemical analysis. If the bunk feeders were discovered to be empty, an additional 10% of the previously supplied amount was added to the next day’s feeding.

Feed bunks were carefully managed to ensure that no more than 10% of feed per animal remained one hour before each feeding. Regular weight measurements of the goats were taken at the study’s outset and subsequently every two weeks in the morning. The feed samples were subjected to various analyses, including dry matter content (determined by heating at 100 °C in an air-forced oven for 24 h, following method 967.03), crude protein (CP, using the Kjeldahl procedure), and ether extract (EE, determined via the Soxtec method using the SXTEC SYSTEM HT 1043 Extraction Unit by TECATOR, based in Hoganas, Sweden) following the AOAC [11] protocols. For the assessment of NDF and ADF, the ANKOM2000 fiber analyzer apparatus (modified to employ the techniques described by Van Soest et al. [12]) was used. These analyses were conducted with the use of heat-stable alpha-amylase and sodium sulfite, and the results were expressed as residual ash content.

### 2.2. Digestion and N Balance Experiment

On the 49th day of the fattening period, five kids were selected at random from each treatment diet and placed into individual metabolic cages, each measuring 1.05 m × 0.80 m. These cages were deliberate to separate urine and feces, facilitating the evaluation of nutritional digestibility and nitrogen (N) balance. A 5-day acclimation period was provided to allow the animals to adapt to their metabolic crates, followed by a 5-day collection phase. Throughout the 5-day collection period, observations were made regarding feed acceptance and rejection. For subsequent analysis, samples of both the feed provided and any refusals were collected. Furthermore, after collecting, weighing, and recording, 10% of the daily fecal output was preserved for later analysis. Urine was also collected, weighed, and recorded in plastic containers, with 5% of the urine being set aside to assess N retention. To prevent ammonia loss, each urine container had 50 mL of 6N HCl (hydrochloric acid) added.

All fecal samples were finely ground to pass through a 1 mm screen (Brabender OHG Duisdurg, Kulturstrasse 51-55, type 880845, Nr 958084, Germany) and subsequently stored for later analysis. Before storage, they were dried at 55 °C in a forced-air oven to ensure consistent weight. In the digestibility and nitrogen balance experiment, the levels of DM, CP, EE, NDF, and ADF in both the feed, refusals, and feces were determined using previously established methods. To assess the nitrogen balance, urine samples were analyzed for N content using the Kjeldahl technique as specified in AOAC [11].

### 2.3. Slaughtering Procedures

At the end of the study, all lambs were slaughtered to evaluate carcass characteristics and meat quality. Following an approximately 18 h fasting period, the animals were processed by experienced personnel using standard slaughter techniques [13]. Subsequently, the carcasses were cooled at 4 °C for 24 h, after which they were reweighed to calculate the dressing percentage, which is the ratio of cold carcass weight to fasting live weight. Final live weight, fasted live weight, and hot carcass weight were also recorded. Immediately after slaughter, non-carcass edible components, including the trachea, lungs, liver, heart, kidneys, spleen, and kidney fat, were separated and weighed.

The next day, the cold carcasses were evaluated using the following methods, as per Abdullah et al.’s work [13]: measurements of the carcass length, limb length, gigot width, breadth behind the shoulders, maximum shoulder width, and tissue depth. The carcasses were then divided into cuts for the shoulders, rack, loin, and legs. This procedure involved further dissecting the loin cut, carefully extracting the longissimus muscle, vacuum-sealing the loin cut, and storing it at −20 °C for two weeks to prepare it for the subsequent assessment of meat quality.

### 2.4. Meat Quality Measurements

Meat quality characteristics, including pH, color (CIE L* a* b* coordinates), shear force, water-holding capacity (WHC), and cooking loss (CL) values, were assessed. The frozen longissimus muscles were stored in plastic bags and thawed overnight in a refrigerator at 4 °C. Each muscle was subsequently sliced to a predetermined thickness, and each slice was employed to evaluate specific meat quality attributes. Slices measuring 15 mm in thickness were utilized for color assessment. These slices were arranged on a polystyrene tray, covered with a breathable film, and allowed to oxygenate for two hours at a temperature of 4 °C.

To assess cooking loss, slices measuring 25 mm in thickness were selected. These slices were initially weighed before the cooking process, placed into plastic bags, and cooked in a water bath at 75 °C for a duration of 90 min. Subsequently, the slices were reweighed after cooking to ascertain the quantity of water lost during the cooking process. As for the cooked slices, they were stored in a freezer at a temperature of 4 °C overnight. Following this, six cores of meat with a diameter of 1 cm were extracted from the slices to calculate the shear force values [14]. To determine the peak force required for shearing the samples, meat cores were sheared perpendicular to the direction of muscle fibers. This was accomplished using a Warner-Bratzler shear blade equipped with a triangular slot cutting edge, which was mounted on a Salter Model 235 (Warner-Bratzler meat shear, G-R Manufacturing Co., 1317 Collins LN, Manhattan, KS, USA). The peak force measurement was conducted on the cooked slices. To measure the pH, a pH spear (large screen, waterproof pH/temperature tester, double injection, model 35634-40, Eurotech Instruments, Shah Alam, Malaysia) was employed. This was carried out by homogenizing 2 g of fresh meat in 10 mL of a neutralized 5 mm iodoacetate solution after thawing. The WHC was determined using a method outlined by Grau and Hamm [15]. Initially, 5 g of raw meat was divided into small pieces and placed between two filter papers and two quartz plates. The meat was then subjected to a weight of 2500 g for a duration of 5 min, after which it was removed and weighed. The water-holding capacity was subsequently calculated as a percentage of the initial weight, following the formula WHC% = [(initial weight − final weight)/initial weight] × 100 [15].

### 2.5. Blood Parameters

Before feeding on days 1, 30, and 60 of the trial, blood samples were taken from each kid through their jugular veins using simple vacutainers at 08:00 h. To separate blood serum, blood samples were centrifuged at 9:00 a.m. Subsequently, serum samples were collected and stored at −20 °C. As per the instructions provided by the manufacturers of spectrophotometer equipment and commercial kits, the blood’s biochemical parameters, such as serum glucose, urea N content, cholesterol, low-density lipoprotein (LDL), high-density lipoprotein (HDL), triglycerides, creatinine, aspartate aminotransferase (AST), alkaline phosphatase (ALP), and alanine aminotransferase (ALT), were measured.

### 2.6. Statistical Methods

The data were analyzed using the MIXED procedure in SAS [16]. In all datasets, the only fixed effect considered was the treatment. In cases where the fixed effects showed significance (*p* ≤ 0.05), appropriate pair-wise t-tests were employed to differentiate the least squares means.

## 3. Results

### 3.1. Nutrients’ Intake and Growth Performance

The intakes of DM, CP, NDF, ADF, and ME were similar (*p* ≥ 0.1137) among the three treatment groups (Table 2). However, kids in the OC150 group had the highest (*p* < 0.0001) EE intake, followed by that of the OC75 and CON groups. The initial BW, final BW, TWG, and ADG of black goat kids were similar (*p* ≥ 0.1257) across the treatment groups. Feed efficiency was not improved by the inclusion of the OC. However, from an economic perspective, the gain cost was decreased (*p* < 0.0039) in the OC diets vs. the CON diet.

### 3.2. Nutrient Digestibility and N Balance

The DM, CP, and ADF digestibility were similar (*p* ≥ 0.1039) among the three treatment groups (Table 3). However, the NDF digestibility improved (*p* = 0.0479) in the OC150 group relative to the OC75 and CON groups. The EE digestibility was greater (*p* < 0.0001) in the OC diets than in the CON diet.

Nitrogen intake was similar (*p* = 0.2551) among the three experimental groups. The fecal N losses were notably reduced (*p* = 0.0331) in kids who received the OC150 and OC75 diets in comparison to those consuming the CON diet. Nevertheless, no differences were detected in N excretion through urine among the three groups. Furthermore, the amount of nitrogen retained per day (g/d) was greater (*p* = 0.0421) in both the OC150 and OC75 groups when contrasted with the CON group. Conversely, the percentage of N retention (g/100 g) was similar across all three groups.

### 3.3. Carcass and Non-Carcass Components

The presence of OC had no discernible effect (*p* ≥ 0.1310) on various parameters, including fasting live weight, cold carcass weight, hot carcass weight, and dressing percentage, as well as non-carcass components and carcass cuts (Table 4). However, the inclusion of OC increased (*p* = 0.0117) intermuscular fat and total fat in comparison to the group of kids fed the CON diet. Despite these fat-related variations, parameters such as loin weight, total lean, total fat, subcutaneous fat, total bone, the meat-to-bone ratio, and the meat-to-fat ratio remained similar (*p* ≥ 0.1099) among the various diets.

Lean carcass dimension characteristics are presented in Table 5. Kids fed OC150 had more leg fat depth (*p* = 0.0158) compared to kids fed the OC75 and CON diets. However, the tissue depth was greater (*p* = 0.0075) in kids fed the OC150 diet than in the OC75 diet, while the tissue depth of kids fed the OC75 diet was similar to that of kids fed CON and OC150 diets. Eye muscle area, rib fat depth, eye muscle width, eye muscle depth, shoulder fat depth, and fat depth were similar among kids fed the three diets.

### 3.4. Meat Quality Characteristics

The values of CL, WHC, and SF were affected by feeding OC (Table 6). Cooking loss was lower in the meat of kids fed OC diets compared to those fed the CON diet, and the WHC value was lower in OC150 compared to the OC75 and CON diets. However, SF was higher in the CON group compared to the OC groups. Feeding OC did not affect (*p* ≥ 0.1543) the meat pH and color coordinates.

### 3.5. Blood Parameters

The impact of OC on the serum metabolite profiles of black goat kids is detailed in Table 7. Among the OC75, OC150, and CON groups, only the activity of the ALT enzyme exhibited differences (*p* < 0.0001) among the three diets. Specifically, the ALT levels were lower in the blood of kids who received the OC75 and OC150 diets when compared to those consuming the CON diet. Conversely, the levels of all other blood serum metabolites, including urea nitrogen, glucose, cholesterol, LDL, HDL, triglycerides, creatinine, ALP, and AST, remained unaffected by the level of OC supplementation.

## 4. Discussion

The growth phase in young goat kids is crucial and necessitates a nutritionally balanced diet, which is often perceived as being relatively costly. In the current study, an examination of the diet ingredients and their chemical composition (Table 1) revealed that the nutrient content of these rations exhibited little to no variation, except in the cases of EE and NDF. Notably, the highest EE content was observed in the OC150 diet, followed by the OC75 and CON diets, which can be attributed to the increased proportion of wheat straw and soybean meal replaced with OC in the diets. A study by Chiofalo et al. [17] indicated that due to its residual oil content, OC possesses a higher digestible EE component compared to other fiber sources. Additionally, the NDF content decreased in the OC75 and OC150 diets compared to the CON diet, likely owing to the higher EE content in the OC diets.

The costs associated with feeding comprise the most substantial portion of total variable expenses for livestock operations, thus exerting an influence on both productivity and profitability [18]. In our current research, the OC150 ration exhibited the lowest feed cost, amounting to 376 USD/ton, with the OC75 ration following closely at 400 USD/ton, and the CON ration at 426 USD/ton. The differences in cost among these rations can be attributed to the higher proportion of soybean meal and wheat straw replaced by OC (Table 1). Consequently, the utilization of OC as an alternative feed for black goats is economically advantageous. This finding aligns with the prior scientific literature that has explored the use of alternative feeds as a means to decrease the expenses associated with traditional animal diets [19,20,21].

In line with our findings, Vargas-Bello-Pérez et al. [22] showed that the feed intake of sheep remained unaffected when their diet included OC at levels of 100 or 250 g/kg. In contrast, Cabiddu et al. [23] noted a reduction in feed intake when OC was included in the diet of lactating cows. However, the intake of EE was the greatest in the OC150 diet, followed by that in the OC75 and CON diets. This can be explained by the highest EE content observed in the OC150 diet, followed by that in the OC75 and CON diet (Table 1).

In the present study, the initial BW, final BW, TWG, and ADG of young black goat kids were consistent among all the treatment groups. The similarity in the kids’ final body weight across the three groups can be attributed to their consistent feed intake levels among all three treatment groups. Similarly, Beken and Sahin [24] observed no discernible impacts on the final BW and TWG of Awassi lambs fed a diet containing 200 g/kg OC mixed with concentrate in comparison to lambs exclusively on a control diet. Likewise, in a study by Aljamal et al. [19], no differences were observed in the final BW, total gains, and ADG of both ewes and lambs when OC was administered at 250 g/kg. However, a study by Mioc et al. [25] reported that feeding a 300 g/kg OC diet for Pramenka lambs resulted in a decreased final BW and a lower ADG when compared to groups consuming either a 150 g/kg OC diet or the control diet. Furthermore, Awassi lambs fed diets containing OC demonstrated an accelerated growth rate, higher final BW, and an increased TWG compared to those fed the commercial diet [8].

In the present study, there was a notable reduction in the cost of gain for kids consuming the OC diets when compared to the CON diet. Alternative feed sources are more cost effective when compared to conventional feeds [17,18,19]. As a result, the reduction in cost per kilogram of gain can be attributed to the overall decrease in the total diet cost due to the inclusion of OC. The utilization of such products not only diminishes diet expenses (Table 1) but also lowers the cost of gain in kids fed OC diets (Table 2), thereby enhancing the practical use of alternative feeds like OC.

Except for EE and NDF, there were no variations in the digestibility of DM, CP, or ADF among the three treatment groups in this investigation. This finding is in agreement with previous research, such as the study conducted by Awawdeh and Obeidat [8], which found no disparities in DM, CP, and ADF digestibility when Awassi lambs were fed diets containing OC. Additionally, Ismail and Obeidat [21] reported similar findings, observing no variations in DM, CP, and ADF digestibility when incorporating sun-dried olive leaves into the diets of Awassi lambs. However, contrasting results were obtained in another study, indicating reduced nutrient digestibility except for EE in diets containing 150 g/kg OC feed in comparison to the commercial diet [6]. Yáñez-Ruiz and Molina-Alcaide [26] demonstrated that the inclusion of two-stage olive cake led to an increase in condensed tannins’ content in the diet and a subsequent decrease in the DM, OM, NDF, ADF, and CP digestibility. Martín García et al. [27] also reported low in vitro digestibility values for both two-stage dried olive cake and olive leaves in goats and sheep, particularly concerning CP.

In our study, no differences were observed in the N balance parameters except for fecal nitrogen losses and the amount of nitrogen retained per day; however, in Obeidat’s study [6], a positive impact on nitrogen balance was observed when the diet contained 150 g/kg of OC. A study by Awawdeh and Obeidat [8] reported no differences in the N balance parameters when Awassi lambs were fed diets containing OC. Moreover, Ismail and Obeidat [21] noted a lack of variations in the N balance parameters when introducing sun-dried olive leaves into the diets of Awassi lambs. Aljamal and co-workers [19] also noted that the nitrogen balance parameters remained unaffected when lactating Awassi ewes were provided with OC at a rate of 250 g/kg in comparison to ewes fed the control diet.

Except for intermuscular fat, total fat, leg fat depth, and tissue depth, the OC inclusion did not affect carcass characteristics. The higher levels of intermuscular fat observed in the OC75 and OC150 groups in this study can be attributed to the higher fat content of the diets containing olive cake in comparison to the CON diet. The results of carcass characteristics in this study are consistent with the previous study carried out by Ozdogan et al. [28], who also reported no differences in carcass weight and yield between lambs that were fed diets containing 125 and 250 g/kg OC. Also, Mioc et al. [25] reported that feeding 150 g/kg OC did not impact carcass weight and dressing percentage. However, when OC inclusion was raised to 300 g/kg, it resulted in a reduction in carcass weight and dressing percentage.

In a previous study conducted by Hamdi et al. [29], it was determined that feeding 280 g/d OC had minimal impact on the carcass and meat quality of Barbarine lambs whether they were fed indoors or reared on improved rangeland. Likewise, OC exhibited no discernible impact on chilled carcass weight and dressing percentage when lambs were fed a concentrate containing 330 g/kg OC [30]. Moreover, a separate study revealed that Awassi lambs fed a diet with OC exhibited comparable dressing percentages and carcass weights in comparison to those consuming a control diet [8]. However, Chiofalo et al. [31] found that the addition of OC to the diet at inclusion rates of 75 and 150 g/kg led to significant increases in the slaughter characteristics and intramuscular fat content of beef cattle.

Regarding the impact of OC on meat quality attributes, the inclusion of OC in this study had a discernible effect only on the values of CL, WHC, and SF. In agreement, Chiofalo et al. [31] found that feeding beef cattle OC at 75 and 150 g/kg reduced cooking loss and improved tenderness. A study by Ozdogan et al. [28] found no important differences in the pH values, color parameters, cooking loss, and tenderness of the longissimus dorsi muscle of lambs. However, they observed that varying the levels of olive cake (125 vs. 250 g/kg) had the effect of increasing the water-holding capacity of the meat. Although the increase in weight between the groups was not statistically significant, the total fat, intermuscular fat, and leg fat depth were higher in the groups fed with OC. This increase in fat, especially intermuscular fat, reflected positively [32] on the characteristics of the meat, such as cooking loss, water-holding capacity, and shear force (tenderness). Similarly, Fiems et al. [33] showed that fat can impact the meat’s juiciness by improving its ability to retain water, lubricating the muscle fibers while cooking, and making the meat softer. Ismail and Obeidat [21] reported that the inclusion of olive leaves in the diet had no discernable impact on carcass and meat quality characteristics. Chiofalo et al. [31] reported that 150 g/kg of OC reduced cooking loss and shear force without altering water content, while a 75 g/kg inclusion had no significant impact on cooking loss, shear force, or water content in beef cattle.

Concerning serum metabolite profiles, we observed that only ALT levels were lower in the blood of kids who received OC75 and OC150 diets. The decline in the activity of this enzyme can be attributed to the influence of antioxidants and polyphenols on the liver [20]. The concentrations of all other blood serum metabolites related to lipids (cholesterol, LDL, HDL, triglycerides), renal function (urea, creatinine), and liver function (glucose, AST, ALP) were unaffected by the inclusion of OC when compared to the CON group. These results suggested that OC had no discernible adverse effects on the health of the goat kids, making it a safe addition to ruminant nutrition.

## 5. Conclusions

This study demonstrated that incorporating OC into the diet of black goats can yield positive and comparable influences on various parameters related to growth performance, carcass attributes, and meat quality. Most importantly, the use of OC reduced the diets’ cost and the cost of gain with no adverse effects on animal health. In parallel, it is suggested that the dietary inclusion of OC at an appropriate level can serve as a valuable alternative to other fiber sources like wheat and barley straw.

## Figures and Tables

**Table 1:** Ingredients and chemical composition of diets fed to black goat kids.

Item	Diet ^1^		
	CON	OC75	OC150
Ingredients (g/kg DM)			
Barley grain	465	475	480
Soybean meal	215	205	200
Wheat straw	300	225	150
Olive cake	0	75	150
Salt	10	10	10
Limestone	9	9	90
Vitamin–mineral premix ^2^	1	1	1
Cost (USD/1000 kg) ^3^	426	400	376
Nutrients			
Dry matter, g/kg	905	908	910
Crude protein, g/kg	159	160	161
Neutral detergent fiber, g/kg	333	326	318
Acid detergent fiber, g/kg	149	147	145
Ether extract, g/kg	9.1	16.7	24.2
Metabolizable energy, Mcal/kg ^4^	2.28	2.26	2.23

^1^ Diets were (1) no olive cake (CON), (2) 75 g/kg olive cake (OC75), and (3) 150 g/kg olive cake (OC150). ^2^ Composition per kg contained the following: vitamin A, 600,000 IU; vitamin D3, 200,000 IU; vitamin E, 75 mg; vitamin K3, 200 mg; vitamin B1, 100 mg; vitamin B5, 500 mg; lysine, 0.5%; DL-methionine, 0.15%; manganese oxide, 4000 mg; ferrous sulphate, 15,000 mg; zinc oxide, 7000 mg; magnesium oxide, 4000 mg; potassium iodide, 80 mg; sodium selenite, 150 mg; copper sulphate, 100 mg; cobalt phosphate, 50 mg; and dicalcium phosphate, 10,000 mg. ^3^ Calculated based on ingredients prices in 2023. ^4^ Calculated based on tabular value of NRC [11] (2007).

**Table 2:** Effects of olive cake (OC) feed on nutrient intake and growth performance of black goat kids.

Item	Diet ^1^				
	CON (n = 10)	OC75 (n = 10)	OC150 (n = 10)	SE	*p* Value
Nutrient intake					
Dry matter, g/d	691	695	723	17.77	0.3445
Crude protein, g/d	110	111	116	2.25	0.1137
Neutral detergent fiber, g/d	230	226	230	2.13	0.4235
Acid detergent fiber, g/d	103	102	105	1.10	0.1306
Ether extract	6.3 ^a^	11.6 ^b^	17.5 ^c^	0.079	<0.0001
Metabolizable energy, Mcal/d	1.57	1.57	1.61	0.015	0.1224
Initial weight, kg	17.0	17.1	17.8	0.40	0.3521
Final weight, kg	24.9	25.9	26.3	0.51	0.1668
Total gain, kg	7.9	8.8	8.6	0.36	0.1536
Average daily gain, g/d	126.1	139.8	134.4	5.72	0.1561
FCR ^2^	5.59	5..02	5.39	0.224	0.1257
Cost (USD/kg gain)	2.38 ^b^	2.01 ^a^	2.03 ^a^	0.090	0.0039

^1^ Diets were no olive cake (CON), 75 g/kg olive cake (OC75), and 150 g/kg olive cake (OC150). ^2^ FCR = Feed Conversion Ratio. Different letters indicate a significant difference at *p* < 0.05.

**Table 3:** Effects of olive cake (OC) feed on nutrient in vivo digestibility and N balance of black goat kids.

Item	Diets ^1^				
	CON (n = 5)	OC75 (n = 5)	OC150 (n = 5)	SE	*p* Value
Digestibility coefficients					
Dry matter	74.4	72.6	71. 9	1.62	0.5538
Crude protein	75.4	73.9	71.4	2.15	0.4252
Neutral detergent fiber	61.30 ^a^	60.5 ^a^	64.4 ^b^	2.18	0.0479
Acid detergent fiber	51.6	54.1	57.9	1.83	0.1039
Ether extract	69.2 ^a^	77.9 ^b^	81.8 ^c^	1.22	<0.0001
N balance					
N intake, g/d	21.6	22.8	23.4	0.72	0.2551
N in feces, g/d	5.6 ^b^	4.3 ^a^	4.2 ^a^	0.35	0.0331
N in urine, g/d	5.2	6.0	6.7	0.45	0.0877
N retained, g/d	10.8 ^a^	12.6 ^b^	12.5 ^b^	0.48	0.0421
Retention, g/100 g	50.1	55.2	53.5	2.04	0.2326

^1^ Diets were no olive cake (CON), 75 g/kg olive cake (OC75), and 150 g/kg olive cake (OC150). Different letters indicate a significant difference at *p* < 0.05.

**Table 4:** Effects of olive cake (OC) feed on the carcass, non-carcass components, carcass cut weights, and loin cut tissues percentages of black goat kids.

Item	Diets ^1^				
	CON (n = 10)	OC75 (n = 10)	OC150 n = 10	SE	*p* Value
Fasting live weight (kg)	24.6	25.6	26.1	0.51	0.1578
Hot carcass weight (kg)	11.8	12.1	12.8	0.35	0.1535
Cold carcass weight (kg)	10.8	11.1	11.7	0.33	0.1825
Dressing percentage	43.6	43.3	44.8	0.63	0.1586
Non-carcass components (kg) ^2^	1.2	1.3	1.3	0.04	0.1900
Carcass cut weights (kg) ^3^	10.3	10.6	11.2	0.31	0.1310
Loin weight, g	361.9	386.5	384.1	12.55	0.3337
Intermuscular fat (g)	12.7 ^a^	20.5 ^b^	19.8 ^b^	1.20	0.0117
Subcutaneous fat (g)	13.6	17.6	18.2	1.57	0.1065
Total fat (g/100 g)	7.2 ^a^	9.9 ^b^	9.8 ^b^	0.63	0.0121
Total lean (g/100 g)	49.4	50.8	50.1	1.28	0.7291
Total bone (g/100 g)	35.1	32.4	31.6	1.67	0.3182
Meat to bone ratio	1.4	1.7	1.6	0.12	0.3103
Meat to fat ratio	7.7	5.3	5.3	0.89	0.1099

^1^ Diets were no olive cake (CON), 75 g/kg olive cake (OC75), and 150 g/kg olive cake (OC150). ^2^ Non-carcass components (Heart, liver, spleen, kidney, lungs, and trachea). ^3^ Carcass cuts (shoulder, racks, loins, and legs). Different letters indicate a significant difference at *p* < 0.05.

**Table 5:** Effects of olive cake (OC) feed on linear dimensions of the longissimus dorsi muscle in black goat kids.

Item	Diets ^1^				
	CON (n = 10)	OC75 (n = 10)	OC150 (n = 10)	SE	*p* Value
Leg fat depth (L3) (mm)	1.00 ^a^	1.05 ^a^	1.30 ^b^	0.070	0.0158
Tissue depth (GR) (mm)	7.50 ^a^	8.30 ^ab^	9.15 ^b^	0.331	0.0075
Rib fat depth (J) (mm)	1.80	1.85	1.85	0.367	0.9929
Eye muscle area (cm^2^)	7.08	7.42	7.34	0.172	0.3525
Eye muscle width (A) (mm)	43.65	45.55	46.05	0.827	0.0714
Eye muscle depth (B) (mm)	18.17	19.05	18.25	0.469	0.3068
Fat depth (C) (mm)	1.00	1.00	1.00	0.017	1.0000
Shoulder fat depth (S2) (mm)	1.10	1.00	1.10	0.082	0.6147

^1^ Diets were no olive cake (CON), 75 g/kg olive cake (OC75), and 150 g/kg olive cake (OC150). Different letters indicate a significant difference at *p* < 0.05.

**Table 6:** Effects of olive cake (OC) feed on the physicochemical properties of the longissimus dorsi muscle in black goat kids.

Item	Diets ^1^				
	CON (n = 10)	OC75 (n = 10)	OC150 (n = 10)	SE	*p* Value
pH ^2^	5.86	5.85	5.85	0.005	0.3160
Cooking loss (g /100 g)	43.01 ^b^	40.11 ^a^	41.15 ^a^	0.579	0.0075
Water-holding capacity (g/ 100 g)	37.63 ^b^	38.08 ^b^	33.08 ^a^	0.860	0.0004
Shear force (kg/cm^2^)	7.89 ^b^	6.53 ^a^	6.84 ^a^	0.363	0.0400
Color coordinates					
L* (whiteness)	31.20	30.17	29.23	0.750	0.1821
a* (redness)	1.57	1.71	1.75	0.101	0.3430
b* (yellowness)	20.25	19.18	18.92	0.610	0.1543

^1^ Diets were no olive cake (CON), 75 g/kg olive cake (OC75), and 150 g/kg olive cake (OC150). ^2^ pH measured after thawing. Different letters indicate a significant difference at *p* < 0.05.

**Table 7:** Effects of olive cake (OC) feed on the blood metabolites in black goat kids.

Item ^2^	Diet ^1^				
	CON (n = 10)	OC75 (n = 10)	OC150 (n = 10)	SEM	*p* Value
Blood urea nitrogen, mg/dL	29.41	30.04	30.06	1.066	0.8867
Glucose, mg/dL	63.33	60.59	61.14	1.718	0.5005
Cholesterol, mg/dL	54.52	55.14	52.21	2.305	0.6428
HDL, mg/dL ^2^	32.26	34.03	33.98	1.400	0.6010
LDL, mg/dL ^2^	19.14	15.92	14.04	1.764	0.1374
Triglycerides, mg/dL	20.56	22.78	20.31	1.420	0.4123
Creatinine, mg/dL	1.02	1.04	1.15	0.056	0.2496
AST, IU/L ^2^	64.70	45.9	48.44	7.701	0.1778
ALT, IU/L ^2^	9.07 ^b^	3.44 ^a^	4.98 ^a^	0.682	<0.0001
ALP, IU/L ^2^	114.29	114.44	116.53	14.178	0.9922

^1^ Diets were no olive cake (CON), 75 g/kg olive cake (OC75), and 150 g/kg olive cake (OC150). ^2^ HDL: high-density lipoprotein; LDL: low-density lipoprotein; AST: aspartate aminotransferase; ALT: alanine aminotransferase; ALP: alkaline phosphatase. Different letters indicate a significant difference at *p* < 0.05.

## Data Availability

The data will be provided upon request from the authors.
